# Cytokine network analysis of immune responses before and after autologous dendritic cell and tumor cell vaccine immunotherapies in a randomized trial

**DOI:** 10.1186/s12967-020-02328-6

**Published:** 2020-04-21

**Authors:** Gabriel I. Nistor, Robert O. Dillman

**Affiliations:** AIVITA Biomedical, Inc., 18301 Von Karman, Suite 130, Irvine, CA 92612 USA

**Keywords:** Proteomics, Principal component analysis, Discriminant analysis, Metastatic melanoma, Dendritic cells, Cancer vaccines

## Abstract

**Background:**

In a randomized phase II trial conducted in patients with metastatic melanoma, patient-specific autologous dendritic cell vaccines (DCV) were associated with longer survival than autologous tumor cell vaccines (TCV). Both vaccines presented antigens from cell-renewing autologous tumor cells. The current analysis was performed to better understand the immune responses induced by these vaccines, and their association with survival.

**Methods:**

110 proteomic markers were measured at a week-0 baseline, 1 week before the first of 3 weekly vaccine injections, and at week-4, 1 week after the third injection. Data was presented as a deviation from normal controls. A two-component principal component (PC) statistical analysis and discriminant analysis were performed on this data set for all patients and for each treatment cohort.

**Results:**

At baseline PC-1 contained 64.4% of the variance and included the majority of cytokines associated with Th1 and Th2 responses, which positively correlated with beta-2-microglobulin (B2M), programmed death protein-1 (PD-1) and transforming growth factor beta (TGFβ1). Results were similar at baseline for both treatment cohorts. After three injections, DCV-treated patients showed correlative grouping among Th1/Th17 cytokines on PC-1, with an inverse correlation with B2M, FAS, and IL-18, and correlations among immunoglobulins in PC-2. TCV-treated patients showed a positive correlation on PC-1 among most of the cytokines and tumor markers B2M and FAS receptor. There were also correlative changes of IL12p40 with both Th1 and Th2 cytokines and TGFβ1. Discriminant analysis provided additional evidence that DCV was associated with innate, Th1/Th17, and Th2 responses while TCV was only associated with innate and Th2 responses.

**Conclusions:**

These analyses confirm that DCV induced a different immune response than that induced by TCV, and these immune responses were associated with improved survival.

*Trial registration* Clinical trials.gov NCT004936930 retrospectively registered 28 July 2009

## Background

The development of effective therapeutic cancer vaccines has been an elusive goal for several decades. The Nobel Prize winning research of Ralph Steinman [1, 2] has led to a resurgence of interest in therapeutic dendritic cell vaccines (DCV) [3, 4], especially in patients with metastatic melanoma [5]. Clinical studies utilizing autologous dendritic cells loaded with antigens from autologous tumor cells have been especially promising [6–9]. A randomized phase II trial tested two vaccines featuring autologous tumor antigens (ATA): injections of autologous dendritic cells loaded ex vivo with antigens from autologous tumor cell lines (DCV), and tumor cell vaccines (TCV) consisting of irradiated autologous proliferating tumor cells [8, 9]. An early analysis showed that DCV was associated with better survival [8], and this was confirmed when 5-year follow up showed a more than doubling of median survival and 3-year survival rate, and a 70% reduction in the risk of death [9]. This DCV approach is currently being tested in phase II trials in glioblastoma and ovarian cancer, and in a phase IB trial in combination with monoclonal antibodies to programmed death-1 protein (PD-1) in melanoma patients.

The human immune response to pathogens and cancer has many interacting components encompassed by concepts of innate and adaptive immunity [10, 11]. Inducing new immune responses, or enhancing existing weak immune responses to cancer associated antigens, is the goal of anti-cancer vaccines [12, 13]. Innate immunity features natural killer cells and associated cytokines [14–17], and macrophages which also produce cytokines and their function is affected by cytokines [18–21]. Adaptive immunity includes Th1 cell-mediate immunity and Th2 humoral immunity [22]. Th1 encompasses cellular responses, notably cytotoxic T lymphocytes and associated cytokines, especially interferon-gamma [23–25]. Th2 encompasses humoral immunoglobulin immune responses with class switching of immunoglobulin isotypes orchestrated by B lymphocytes with the assistance of helper T lymphocytes, and these responses are associated with various cytokines [26–30]. There is also a Th17 response that features cells helper T cells that secrete IL17 [31–33]. These cells can augment immunosuppressive or immune enhancing effects depending on the microenvironment and local cytokines [34–37]. At the nexus of innate and adaptive immunity, are antigen-presenting cells, especially dendritic cells [38], that are characterized by secretion of IL12 when activated [39–44]. As part of maintaining homeostasis of the immune system, there are also a number of cytokines including such as PD-1 [45, 46], IL10 [47–49], transforming growth factor beta (TGFβ) [50–52], beta-2-microglobulin (B2M) [53, 54], and Fas (CD95) [55], that are associated with suppressed immune responses. In terms of cytokines, IL17 [34, 36, 37], and IL18 [56, 57], can be immune-augmenting or immunosuppressive depending on other signals in the microenvironment.

One of the objectives of vaccine clinical trials is to increase understanding of the immune responses that are induced or enhanced by such vaccines and correlating these responses with survival. In the randomized phase 2 analysis of 110 cytokines showed that variation in specific cytokine groupings were similar at baseline but differed markedly following three vaccinations [9]. A combination of baseline soluble programmed death protein-1 (sPD-1) and changes in sPD-1 after three injections was strongly predictive of 3-year survival in DCV-treated patients, but not TCV-treated [10]. The current analysis was conducted in an effort to better understand the nature of these immune responses in terms of classical concepts of innate and adaptive immune responses as reflected by changes in cytokines in response to vaccine therapy [58].

## Methods

### Serum samples

Blood samples from melanoma patients enrolled in a randomized phase 2 trial (clinicaltrials.gov NCT00436930) were obtained at week-0, 1 week before the first of 3 weekly injections of DCV or TCV vaccines, and week-4, 1 week after the third weekly injection [8, 9]. The trial was approved by the Western Institutional Review Board (Seattle, WA., WIRB^®^ Protocol #20090753). Patients gave written informed consent for randomization to DCV or TCV, and blood collection and analysis. The protocol and manufacturing procedures were reviewed by the US Food and Drug Administration (BB-IND 5838 and BB-IND 8554). TCV consisted of irradiated autologous tumor cells from a short-term cell line; DCV consisted of autologous dendritic cells incubated with irradiated autologous tumor cells [8, 9]. Both DCV and TCV were admixed in granulocyte-macrophage colony stimulating factor (GM-CSF) just prior to subcutaneous injections scheduled for weeks 1, 2, 3, 8, 12, 16, 20, and 24.

### Analysis of serum markers

Cryopreserved 200-microliter serum samples from week-0 and week-4 were analyzed for 110 cytokines, growth factors, proteases, soluble receptors and other proteins as shown in a supplementary table of a previous publication [9], using a quantitative, multiplex enzyme-linked immunosorbent assay (Quantibody^®^ Cytokine Array, Raybiotech, Inc., Norcross, GA.). Values were expressed as absolute concentration (pg/mL) and as percentage differences above or below the mean value from three normal controls.

### Principal component analysis

Principal component analysis (PCA) was performed using IBM SPSS Statistics V26. PCA transforms data into a coordinate system by creating new uncorrelated variables that successively maximize variance [59, 60]. The most important use of PCA is to represent a multivariate data table as smaller sets of variables (summary indices) in order to observe trends, jumps, clusters and outliers. PCA is valuable for exploratory analysis of extensive data and especially useful for integrating genomics and proteomic datasets in effort to understand biological processes [61–63].

In a two-component PCA model variables are distributed in a two-dimensional plane so that the largest amount of variance is grouped in principal component-1 (PC-1) along one axis and the next highest is grouped in principal component-2 (PC-2) along an orthogonal axis. The distribution is based on correlation coefficients that vary from 0 to 1 or 0 to − 1 from the origin, which quantitates the strength of positive or negative correlation. Positively correlated variables cluster in the same side of at least one of the components (influential), or in the same quadrant (correlated). Negatively correlated variables are positioned on opposite sides of the origin in diagonally opposed quadrants. After plotting, the component axes can be rotated for optimal delineation of the groups, following established procedures (e.g. Varimax, Quartimax, Promax, etc.) that give a best fit among variables. The rotation changes only the positioning vector of the components to each-other for easier interpretation.

The Kaiser–Meyer–Olkin Measure of Sampling Adequacy (KMO) was used to indicate the proportion of variance that might be attributed to specific variables. High values (close to 1.0) indicate that a factor analysis of the data will be useful as opposed to values less than 0.50. Bartlett’s Test of Sphericity (BTS) was used to test the hypothesis that the correlation matrix is an identity matrix, which would indicate that variables are unrelated and therefore unsuitable for structure detection. Lower significance levels (less than 0.05) indicate that a factor analysis of the data may be useful.

### Discriminate analysis

Discriminant analysis (DA) was used to interpret multiple discriminant functions arising from analyses involving more than two groups and more than one variable [64]. DA is very useful for detecting variables that discriminate between different groups and can classify cases into different groups with a better than chance accuracy. It is especially useful for interpreting large data bases such as result from proteomics [65–67]. The objective is to develop discriminant functions that are the linear combination of independent variables (i.e. change of cytokines) that will discriminate between the categories of the dependent variable (i.e. survival groups). It enables one to examine whether significant differences exist among the groups based on the predictor variables and evaluates the accuracy of the classification. Several variables are included in order to see which one(s) contribute to discriminating between groups, and then a matrix of total variances and covariances is created as well as a matrix of pooled within-group variances and covariances. Matrices are compared via multivariate F tests to determine whether there are significant differences (with regard to all variables) between groups. This procedure is identical to multivariate analysis of variance, or MANOVA.

In stepwise discriminant function analysis, a model of discrimination is built step-by-step. At each step all variables are reviewed and evaluated to determine which one contributes most to discriminating between groups. That variable is included in the model, and the process starts again. The stepwise procedure is “guided” by F-to-enter and F-to-remove values for statistical-based discrimination between groups; i.e., it is a measure of the extent to which a variable makes a unique contribution to the prediction of group membership. When performing a multiple group discriminant analysis, an optimal combination of variables is automatically determined so that the first function provides the most discrimination between groups, the second provides second most, and so on. The larger the standardized coefficient, the greater the variable’s contribution to discrimination. Another way to determine which variables define a particular discriminant function is to examine the factor structure coefficients, which are the correlations between variables in the model and the discriminant functions. A Bonferroni correction of p values was used in analyses that included multiple comparisons.

### Principal cytokine pathway analysis

The pathway analysis considered the findings from both PCA and DA. The methods to investigate the effect of these pathways on survival included linear regression analysis and tests of equality of group means (univariate ANOVA). In order to interpret multiple discriminant functions arising from analyses with more than two groups and more than one variable, we first tested the different functions for statistical significance, and only considered significant functions for further examination. Next, we looked at the standardized b coefficients for each variable for each significant function. The larger the standardized b coefficient, the larger is the respective variable’s unique contribution to the discrimination specified by the respective discriminant function. The classification matrix was used to determine how well the current classification functions predict group membership as defined by survival. The classification matrix shows the number of cases that were correctly classified (on the diagonal of the matrix) and those that were misclassified. To perform the discriminant analysis each treatment arm was sub-divided into three categories: survivors over 60 months, and the remaining patients split at the median survival for each treatment arm.

## Results

Paired week-0 and week-4 blood samples were available for 22 of 24 TCV-treated patients and 17 of 18 DCV-treated patients [9]. The two missing TCV-treated patients did not have a week-4 sample because of rapidly progressing metastatic disease and both died within 2 months of enrollment. The missing DCV-treated patient was alive at 5 years but had rescinded permission to study his blood samples.

### Principal component analysis at baseline

**Fig. 1:**
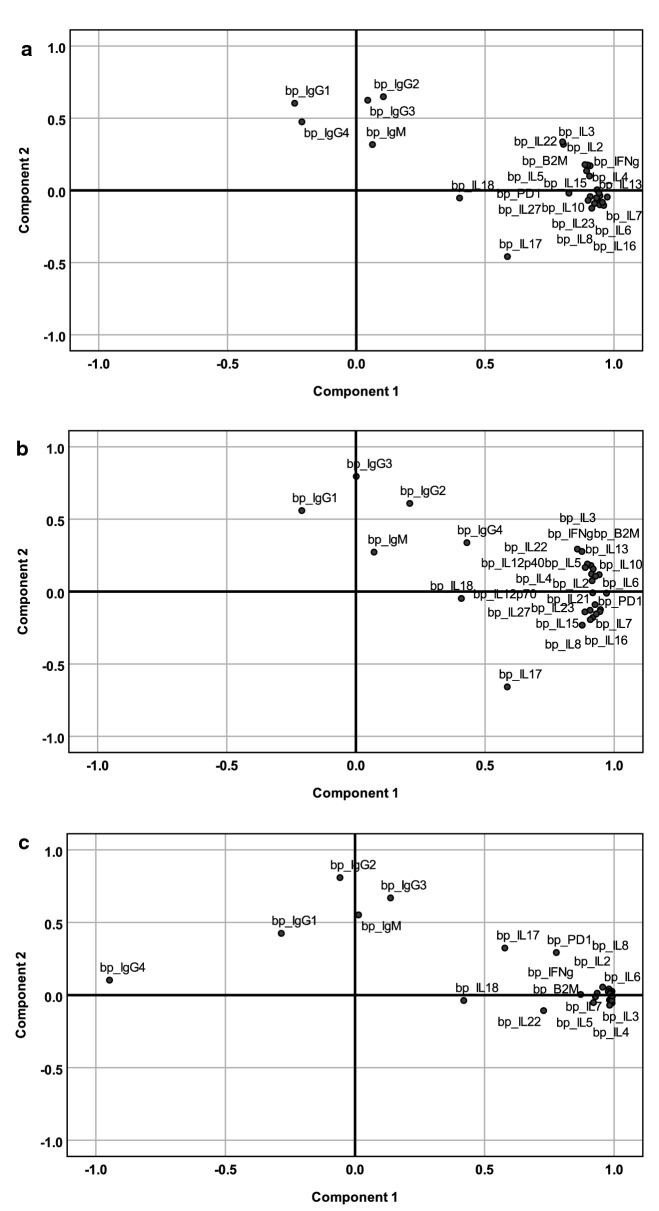
Principal component analysis of baseline serum cytokine levels. Component loading plot of baseline serum protein levels for (**a**) all 39 patients regardless of treatment arm (n = 39). (KMO = 0.719; BTS < 0.001), At baseline the 22 TCV-treated patients (**b**) and the 17 DCV-treated patients had similar, distribution of cytokine associations

The PCA of cytokines and immunoglobulins (Ig) at baseline are shown in Fig. 1. Before vaccine treatment was initiated, the distribution of PCA values for TCV-treated and DCV-treated patients were similar. Deviation from normal values for all 39 patients defined two distinctive groups based on the loading plot. The correlative group of cytokines on component 1 (PC1) accounted for 64.4% of variance and a correlative group of immunoglobulins (PC2) accounted for 7.5% of variance (Additional file 1). As shown in Table 1, the majority of the cytokines with correlations greater than 0.5 are those classically associated with Th1, and Th2 immune responses.

**Table 1:** PCA component matrix for various cytokines at baseline

Cytokine	Component 1	Component 2	Cytokine	Component 1	Component 2
IL13	*.974*	− .046	IL27	*.900*	− .069
IL23	*.961*	− .104	IL2	*.898*	.176
TGFβ1	*.955*	− .083	B2M	*.894*	.135
IL6	*.946*	− .037	IL12p40	*.887*	.178
IL7	*.945*	− .100	PD1	*.825*	− .019
IL12p70	*.942*	− .019	IL3	*.804*	.319
IL15	*.935*	− .063	IL22	*.801*	.336
IL4	*.934*	.004	IL17	*.587*	− .459
IL21	*.933*	− .052	IL18	.401	− .053
IL16	*.925*	− .091	IgG2	.105	*.649*
IL8	*.914*	− .123	IgG3	.044	*.624*
IL10	*.908*	− .043	IgG1	− .239	*.604*
IFNγ	*.908*	.171	IgG4	− .211	.475
IL5	*.905*	.099	IgM	.063	.318

Variables with strong correlative distribution (> 0.5) are in italic

This collection of cytokines positively correlated with B2M, PD1 and TGFβ1 suggesting coexistence of pre-existing tumor-associated inflammation and immunosuppression.

### Principal component analysis of cytokine changes after 3 weekly injections

**Fig. 2:**
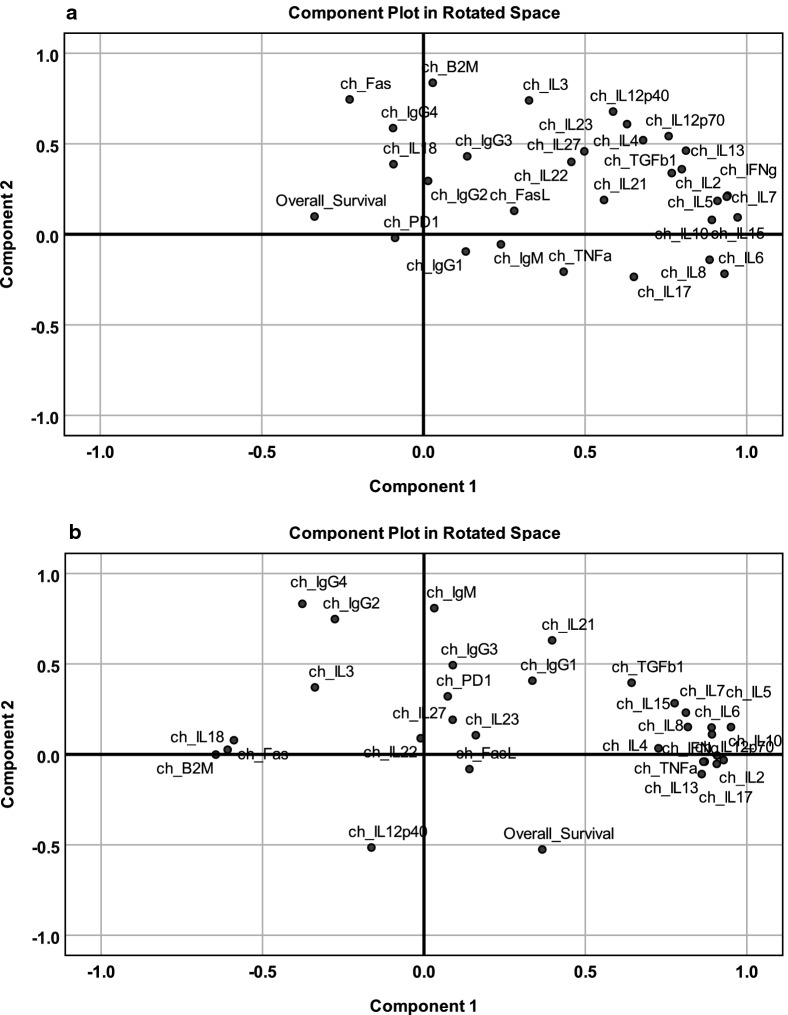
Principal component analysis of cytokine changes after 3 weekly vaccine injections. After 3 injections there was a noticeable difference in the distribution of cytokines between **a** TCV-treated patients and **b** DCV-treated patients with much tighter clustering in component 1 of the DCV-treated patients

Figure 2 shows the PCA of cytokine changes after 3 weekly vaccine injections. The change from baseline for each cytokine was calculated by subtracting the baseline value from the week-4 value and dividing the difference by the smaller of the baseline or the week-4 value to avoid negative numbers when week-4 levels were lower than baseline values. Using this method, a positive or negative correlation vector was calculated for each variable.

### Cytokine changes after 3 TCV injections

In TCV-treated patients, PCA showed the contribution of multiple factors with the combination of PC1 and PC2 responsible for only 54% of variance (Fig. 2a, Additional file 2). There was a positive correlation on PC1 for most of the cytokines suggesting the evolution of tumor-associated inflammation, and on PC2 for tumor markers (B2M, FAS receptor) along with Th2-associated cytokines (Fig. 2a, Table 2). Changes in IL12 correlated with both Th1- and Th2-cytokines suggesting an adaptive response mediated by antigen presenting cells (APC) that had been suppressed by TGFβ1. Thus, after three injections, the cytokine changes in the TCV group were most consistent with a Th2 adaptive response and an innate response.

**Table 2:** PCA component matrix for changes in cytokines after three TCV injections

Cytokine	Component 1	Component 2	Cytokine	Component 1	Component 2
_IL15	*.972*	.094	IL4	*.679*	*.520*
IL2	*.941*	.214	IL17	*.651*	− .235
IL7	*.938*	.208	IL23	*.630*	.609
IL6	*.931*	− .218	IL21	*.559*	.190
IL5	*.909*	.185	IL27	.498	.458
IL10	*.892*	.080	OS	− .337	.099
IL8	*.885*	− .140	B2M	.029	*.837*
IL13	*.812*	.462	Fas	− .228	*.745*
IFNγ	*.799*	.360	IL3	.327	*.740*
TGFβ1	*.768*	.338	IL12p40	.587	*.678*
IL12p70	.*758*	*.542*	IgG4	− .094	*.587*

Rotated Component Matrix (Varimax with Kaiser Normalization). Strong correlation values are in italic

*OS* overall survival

PCA limited to factors with component values above 0.5 (Table 3), revealed a significant association of the variables KMO = 0.701, BTS < 0.001 (Additional file 3). The first two components contributed equally (40.1% and 39.5% of the variance respectively (Additional file 4), thus organizing the variables into two groups. One group contains variables associated with IL6, IL15, and IFNγ, but not associated with the APC-associated cytokine IL12p70, while the other group contains markers associated with APCs. This suggests an adaptive Th2 response mediated through immunoglobulins, and possibly a suppressed Th17/Th1 response based on the association with TGFβ1. Survival correlated negatively with the suppressed Th1/Innate response, but slightly positively with the increased immunoglobulins, suggesting dominance of a Th2 response in TCV-treated patients. TCV-treated patients exhibited changes in Th2 cytokines, suggesting that antigens on the injected irradiated tumor cells primarily elicited a Th2 response mediated by endogenous APCs.

**Table 3:** PCA component matrix for changes in cytokines after three TCV injections

Cytokine	Component 1	Component 2	Cytokine	Component 1	Component 2
IL6	*.908*	.214	IL21	*.567*	.285
IL15	*.839*	.499	IL12p40	.202	*.906*
IL5	*.811*	.491	IL23	.252	*.889*
IL17	*.797*	− .045	IL12p70	.456	*.827*
IL10	*.782*	.438	IL4	.375	*.785*
IL2	*.758*	*.597*	IL27	.107	*.759*
IL7	*.731*	*.615*	IL13	*.574*	*.742*
IFNγ	*.650*	*.589*	TGFβ1	*.570*	*.622*

Rotated Component Matrix (Varimax with Kaiser Normalization). Only components greater than 0.5 are shown. Strong correlation values are in italic

### Cytokine changes after 3 DCV injections

In DCV-treated patients there was a correlation between Th1/Th17 cytokines on PC1 and immunoglobulins on PC2 (Fig. 2b) with PC1 and PC2 accounting for 39.1% and 12.6% of variance respectively (Additional file 5). The first two components were used for further analysis and component plotting (Table 4).

**Table 4:** PCA component matrix for changes in cytokines after three DCV injections

Cytokine	Component 1	Component 2	Cytokine	Component 1	Component 2
IL5	*.951*	.151	IL4	*.726*	.034
IL12p70	*.928*	− .032	B2M	*− .645*	− .001
IL10	*.908*	− .006	TGFβ1	*.643*	.396
IL17	*.907*	− .053	IL18	*− .608*	.026
IFNγ	*.892*	.111	Fas	*− .589*	.078
IL6	*.891*	.149	IgG4	− .377	*.833*
IL2	*.870*	− .040	IgM	.032	*.809*
TNFα	*.865*	− .040	IgG2	− .276	*.748*
IL13	*.860*	− .109	IL21	.397	*.631*
IL8	*.817*	.151	OS	.366	*− .526*
IL7	*.811*	.231	IL12p40	− .163	*− .515*
IL15	*.776*	.283			

Rotated Component Matrix (Varimax with Kaiser Normalization). Only components greater than 0.5 are shown. Strong correlation values are in italic

*OS* overall survival

Inflammatory cytokines correlated strongly with an APC-driven effect (IL12p70) through Th1 (IFNγ, TNFα) combined with a Th17 (IL17) response, as well as through Th2 driven by IL12p70 in association with IL5 (hypersensitivity) and immunoglobulins on component 2. Survival correlated with the Th1/Th17 factors on PC1. On both PC1 and PC2, survival correlated positively with IgG1 and IgG3 but negatively with IGM, IgG2 and IgG4. The inverse correlation of IgG4/IgG2 with IgG1/IgG3 is consistent with immunoglobulin class-switching suggesting that antigen-specific immunoglobulin responses may have contributed to survival. It is also noteworthy that B2M and FAS receptor are negatively correlated with the immune response and survival. Collectively the data suggests an adaptive response in the DCV-treated group that included immunoglobulin class-switching and a Th17/Th1 response. The cytotoxic Th17 response is not correlated with IL23 [68, 69], a cytokine that is expected to drive naïve CD4+ cells towards Th17 lineages associated with immunosuppression. Instead, IL17 is strongly correlated with TNFα and IFNγ, consistent with conversion of an existing cognate population of Th17 cells from a tolerizing to a cytotoxicity-facilitating phenotype [34, 36, 37].

Closer examination of factors positively correlated on PC1 confirms a significant association of this group, KMO = 0.718 and BTS < 0.001 (Additional file 6). The analysis of the reduced set of factors identifies two components that account for 49.0% and 33.3% of the variance. Within component 1 IL17 has the highest coefficient, associated with a Th1 response driven by APC (IL12p70) (Table 5). The second component contains the innate response associated with Th2 (IL4, TGFβ1) and other pleiotropic factors (IL7, IL8) (Table 5). Both components were associated with IFNγ and TNFα. Thus, after three DCV-injections, the analysis suggests a multifaceted response driven by the Th1/Th17 cytotoxic (Th1-like) pathway and a Th2 immunoglobulin response.

**Table 5:** PCA component matrix for changes in cytokines after three DCV injections

Cytokine	Component 1	Component 2	Cytokine	Component 1	Component 2
IL17	*.884*	.285	TNFα	*.741*	.479
IL10	*.862*	.377	IFNγ	*.705*	*.557*
IL13	*.859*	.270	IL4	*.539*	*.516*
IL5	*.852*	.459	TGFβ1	.239	*.884*
IL2	*.849*	.291	IL15	.362	*.869*
IL12p70	*.803*	.480	IL7	.390	*.858*
IL6	*.763*	.484	IL8	.497	*.728*

Rotated Component Matrix (Varimax with Kaiser Normalization). Only components greater than 0.5 are shown. Strong correlation values are in italic

### Discriminant analysis by treatment arm and survival

Each treatment arm was sub-divided into three categories based on overall survival (Table 6).

**Table 6:** Survival subgroups defined by survival in each treatment arm

TCV survival groups (n = 22)	TC low	TCV intermediate	TCV high
Number of cases	9	8	5
Median overall survival (months)	9	31	60

DCV survival groups (n = 17)	DCV low	DCV intermediate	DCV high
Number of cases	6	6	5
Median overall survival (months)	15	43	60

*TCV* tumor cell vaccine, *DCV* dendritic cell vaccine

**Fig. 3:**
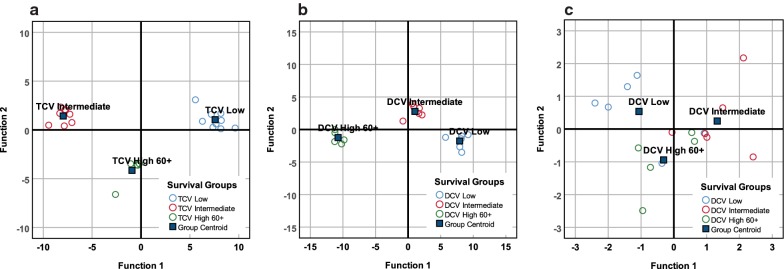
Discriminant analysis of changes in cytokine levels after three injections and survival groups. Distribution of TCV survival groups (**a**) using discriminant analysis of inflammatory cytokines (Wilks’ Lambda p = 0.015). The function coefficients accurately classified all 22 patients into the appropriate survivor subgroup: 9 in the low survival group, 8 in the intermediate survival group, and 5 in the 60+ month survival group. Distribution of DCV survival groups (**b**) using discriminant analysis of inflammatory cytokines (Wilks’ Lambda p = 0.020). The function coefficients accurately classified the survivor subgroups: 6 in the low survival group, 6 in the intermediate survival group, and 5 in the 60+ month survival group. Distribution of DCV survival groups (**c**) discriminant analysis using the most powerful discriminators, TGFβ1 and IL17 (Wilks’ Lambda p = 0.003). The function coefficients accurately classified 70.6% (12/17) in the appropriate survivor subgroups: 4 of 6 in the low survival group, 5 of 6 in the intermediate survival group, and 3 of 5 in the 60+ month survival group

DA was then applied to cytokine data for each patient in each treatment arm with results as displayed in Fig. 3. Regardless of treatment DA correctly classified patients into their appropriate survival subgroup (Additional file 7).

### Discriminant analysis of TCV-treated patients

DA based on changes of inflammatory markers among TCV-treated patients yielded a discriminant function for survival (p = 0.015, Additional file 8 that accounted for 100% of variance with 90.2% of variance in the first discriminant function (Additional file 9). DA also accurately classified the three survivor subgroups (Fig. 3a). However, none of the variables reached statistical significance (Additional file 10). Stepwise DA failed to identify any significant variables qualified for further analysis based on F function entry criteria (p < 0.05). Thus, DA of TCV patients was unable to identify a cytokine pattern to explain the survival distribution.

### Discriminant analysis of DCV-treated patients

DA based on changes of inflammatory markers among DCV-treated patients yielded a discriminant function for survival (p = 0.020, Additional file 11) that accounted for 100% of variance with 92.9% of variance in the first discriminant function (Additional file 12). DA accurately classified the three survivor subgroups (Fig. 3b). Stepwise DA identified IL17 and TGFβ1 as the most important variables that discriminated among survival. These two variables identified two significant discriminant functions (p = 0.003 and p = 0.023, Additional file 13) that explained 100% of variance with 73.1% in the first function (Additional file 14). The discriminant function of TGFβ1 and IL17 correctly classified 70.6% of the DCV-treated patients (Fig. 3c), All coefficients were less than 0.05 for each variable and each survival group (Table 7).

**Table 7:** Changes in IL17 and TGFβ1 and association with survival

	DCV low (n = 6)	DCV intermediate (n = 6)	DCV high 60+ (n = 5)
Change_IL17	− .024	.006	.001
Change_TGFβ1	− .003	.015	− .021
(Constant)	− 2.353	− 1.517	− 1.830

Thus, persistence of TGFβ1 and a decrease in IL17 was associated with relatively short survival. Persistence or increase of IL17 and an increase of TGFβ1 (consistent with an ongoing response that could be either anti-tumor or tolerizing depending on the TGFβ1 dominance) was associated with intermediate survival. A decrease in TGFβ1 combined with an increase of IL17 was associated with long-term survival.

### Immunoglobulins (Th2 pathway)

**Fig. 4:**
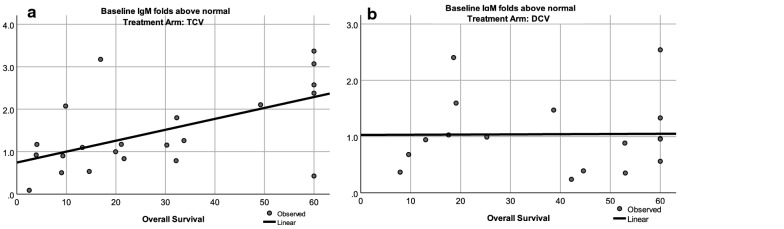
Correlation between baseline IgM and survival. **a** TCV-treated patients, p = 0.006 by ANOVA; **b** DCV-treated patients, p = 0.970 by ANOVA)

PCA suggested there was an existing immunoglobulin response at baseline, but also a new immunoglobulin response in each treatment arm after three injections; therefore, this pathway was examined relative to survival. Baseline immunoglobulin values other than IgM were moderately elevated compared to healthy controls. We assumed that the vaccines were inducing a new response; so, we analyzed changes in IgM that might be expected after exposure to new antigens. The correlation between baseline IgM levels and survival is shown for TCV-treated patients in Fig. 4a, and for DCV-treated patients in Fig. 4b. The average IgM of healthy controls was 120.2 mg/dL, which is in the 40–230 mg/dL standard range of normal IgM for ages 45 years and older [70]. The changes in mean and median Ig levels after three injections of either vaccine are also shown in Table 8.

**Table 8:** Baseline immunoglobulin level at baseline and after 3 injections

	TCV		DCV	
	Mean	Median	Mean	Median
Fold difference compared to normal values				
IgG1	2.15	1.05	2.24	1.43
IgG2	1.99	.66	2.15	.98
IgG3	3.36	2.95	4.28	3.74
IgG4	2.88	2.56	2.56	2.37
IgM	1.47	1.16	1.04	.96
Fold difference compared to baseline after 3 injections				
IgG1	− 1.24	− 0.47	− 0.6.5	0.01
IgG2	− 0.74	0.62	− 0.14	− 0.12
IgG3	− 0.3.1	0.30	− 0.12	0.03
IgG4	− 0.18	− 0.17	0.67	− 0.12
IgM	0.09	0.05	0.32	0.19

The slightly increased baseline IgM in TCV-treated patients correlated with longer survival (Fig. 4a). The baseline IgM level was normal in DCV-treated patients and not correlated with survival (Fig. 4b). Linear regression analysis was confirmed by Cox’s proportional hazards model for survival using baseline IgM as a cofactor in TCV-treated patients (p = 0.014, Additional file 14). After three injections average IgM increased by 31.5% in the DCV-treated patients (Table 8) with 11 of 17 patients having above-normal values. This suggests that a new IgM response was induced in DCV-treated patients (p = 0.102, Wilcoxon signed rank test, p = 0.090 paired t-test).

The DCV regression graph shows two distinct distributions on either side of 36 months (Fig. 4b). This suggests that long-term survivors may have different mechanisms of response; therefore, we analyzed patients who survived less than or more than 60 months separately in both treatment arms (Table 9).

**Table 9:** Association between immunoglobulin levels and survival

	TCV < 60 (n = 17)		TCV 60+ (n = 5)		DCV < 60 (n = 12)		DCV 60+ (n = 5)	
	Mean	Median	Mean	Median	Mean	Median	Mean	Median
Fold difference from normal values at baseline								
IgG1	1.55	1.12	4.17	.98	1.62	1.41	3.75	1.73
IgG2	1.89	.49	2.33	1.09	2.49	.89	1.33	1.05
IgG3	3.55	3.25	2.72	2.53	4.99	4.09	2.58	3.01
IgG4	3.09	2.84	2.14	1.78	2.57	2.33	2.55	2.37
IgM	1.21	1.10	2.36	2.57	0.95	0.91	1.27	0.96
Fold difference compared to baseline after three injections								
IgG1	− 1.32	− 0.48	− 1.00	− 0.05	0.22	0.03	− 0.74	− 0.31
IgG2	− 0.10	0.10	− 0.29	− 0.63	0.04	0.02	− 0.58	− 1.06
IgG3	0.04	0.05	− 0.28	0.18	− 0.12	− 0.08	− 0.12	0.09
IgG4	− 0.15	− 0.14	− 0.28	0.50	0.25	− 0.04	− 0.38	− 0.35
IgM	0.080	0.05	0.17	0.04	0.43	0.24	0.04	− 0.15

TCV-treated patients who survived more than 60 months had elevated baseline IgM levels (p = 0.013, ANOVA) that were higher than for TCV-treated patients who survived less than 60 months (p = 0.033 independent sample t-tests, 0.034 Mann–Whitney), higher than for DCV-treated patients who survived less than 60 months (p = 0.008 independent sample t-tests, 0.027 Mann–Whitney), but not higher than in DCV-treated patients who survived 60+ months (p = 0.183 independent sample t-tests, p = 0.175 Mann–Whitney). Perhaps patients who survived 60+ months had an existing anti-tumor Th2 response that contributed to better survival even in the absence of vaccine treatment. After three injections, only DCV-treated patients who survived less than 60 months had an increase in IgM (p = 0.045 paired t-test, p = 0.06 Wilcoxon signed rank), but this did not correlate with survival (Additional file 15).

### T-helper cytotoxic pathways (Th1, Th17 pathways)

The Th17 phenotype is induced by a combination of IL6, IL23 and TGFβ1 [31–33], and functionally by the amplitude of TGFβ1 [71]. Th17 cells have a dual-state plasticity; a high TGFβ1 induces regulatory or immune-tolerizing effects while low TGFβ1 allows a Th1-like cytotoxic response that includes IFNγ and TNFα [34, 36, 37]. A strong IL12/IL23 response leading to an IL4-mediated response suggests a new T-helper (CD4) response with a new Th17 component [42]. The lack of association with IL23 suggests a helper response that does not require Th17 [42]. Positive correlation with IFNγ suggests a Th1 response; positive correlation with IL10 suggests a Th2 response; and positive correlation with TGFβ1 suggests a regulatory T lymphocyte (Treg) response. In the tumor microenvironment Th17 cells are likely antigen-cognate and can change from a suppressive to cytotoxic state in response to local factors [36, 37]. The sudden increase of IL17 in association with TNFα suggests a pro-inflammatory switch of Th17 cells to a cytotoxic helper function rather than suppression. An IL23 increase could reflect lineage stimulation from naïve CD4+ cells that can be directed to both cytotoxic and regulatory pathways, depending on the local environment. Downstream effectors of Th17 can result in cytotoxic lymphocytes (Th1-like pathway) [36, 37], a Th2 pathway through IgM induction [72], or suppressive (as in a Treg pathway) [32].

**Fig. 5:**
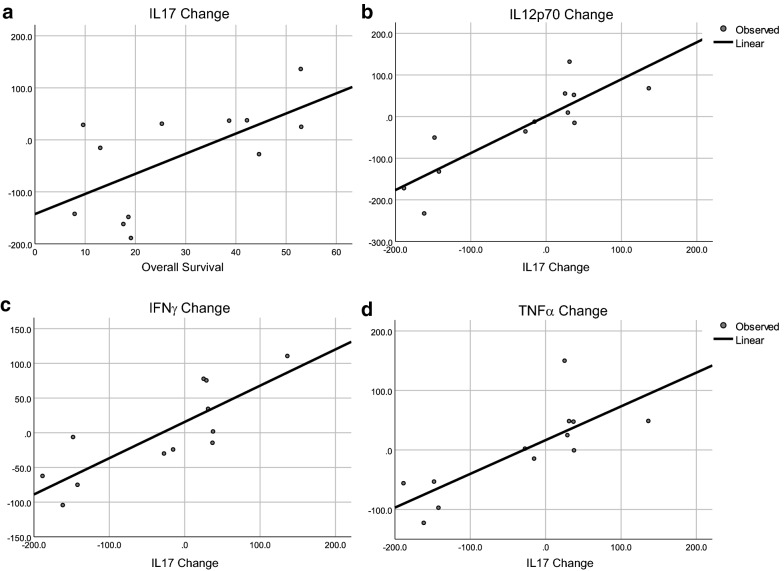
Linear regression analyses of selected cytokines with overall survival. **a** IL17 and survival in DCV-treated patients who survived less than 60 months, p = 0.028 by ANOVA; **b** IL12p70 and IL17 in DCV-treated patients who survived less than 60+ months, p < 0.0001 by ANOVA; **c** IFNγ and IL17 in DCV-treated patients who survived less than 60+ months, p = 0.001 by ANVOVA, and **d** TNFα and IL17 in DCV-treated patients who survived less than 60+ months, p = 0.003 by ANOVA

Because Th17 is a versatile cell type, estimations of Th17 function should include additional factors such as IFNγ, TNF, TGFβ1, and Th17-associated cytokines (IL17, IL21, IL22, IL27, IL31, IL33). All patients were included in linear regression analysis between cytokines and survival, but long-term follow up ended at 60 months; therefore, patients surviving longer were excluded from linear regression analysis of the cytokines selected as impacting survival by PCA and DA. Cytokines identified by DA accurately classified patients into survival groups. The associations between survival, IL17, IL12p70, IFNγ, and TNFα are shown in Fig. 5. The change in IL17 correlated with survival for DCV-treated patients who survived less than 60 months (Fig. 5a). Increases in IL17 were correlated with increased IL12p70, a cytokine produced by DCs (Fig. 5b). IL17 also correlated with IFNγ (Fig. 5c) and TNFα (Fig. 5d), both of which are components of Th1 responses. This suggests that the increased IL17 is associated with a Th1-like response that was triggered by antigen-loaded DC that secreted IL12. To explore the source of IL17, we investigated its association with IL23, another cytokine that can generate IL17-secreting Th17 lymphocytes [73, 74]. IL17 did not correlate with IL23 (p = 0.248, ANOVA); so, the source of IL17 was not a new population of Th17 cells, but possibly resulted from conversion of an existing antigen-cognate Th17 population from a tolerizing-state to a cytotoxicity-inducing state [34, 36, 37].

**Fig. 6:**
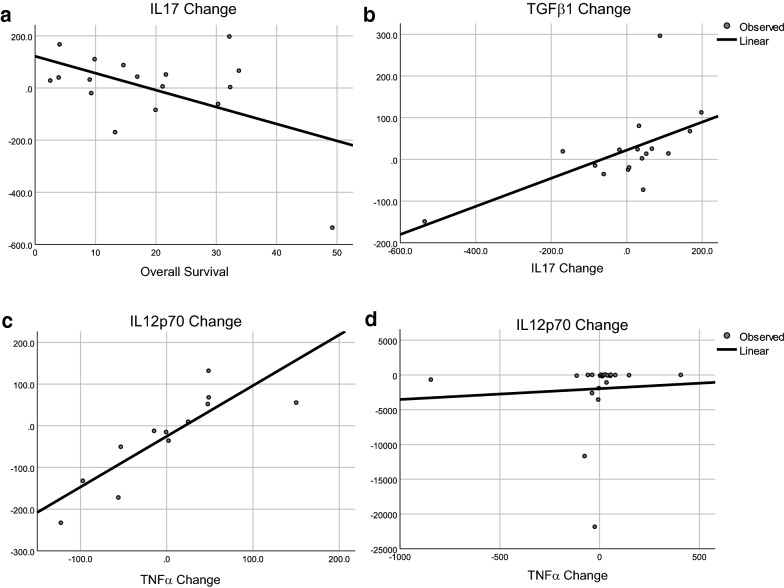
Linear regression for selected cytokines and survival by treatment. **a** Changes in IL17 and survival in TCV-treated patients who survived less than 60+ months, p = 0.034 by ANOVA; **b** changes in TGFβ1 and IL17 in TCV-treated patients who survived less than 60+ months, p = 0.012 by ANOVA; **c** changes in IL12p70 and TNFα after three DCV injections, p < 0.001 by ANOVA; **d** changes in IL12p70 and TNFα after TCV treatment, p = 0.776 by ANOVA

In the TCV arm there were no post-treatment changes in cytokines that correlated with survival except for IL17, which correlated negatively (Fig. 6a). In TCV-treated patients IL17 positively correlated with TGFβ1 (Fig. 6b), and IFNγ (p = 0.038) and cytokines associated with innate immune responses: IL15 (p = 0.003), IL8 (p = 0.002) or Th2-associated cytokines: IL13 (p = 0.038), IL10 (p = 0.024), IL7 (p = 0.038), IL6 (p = 0.01) IL5 (p = 0.07) and IL2 (p = 0.018). The cytokine changes were not consistent with a cytotoxic Th1 response, but rather with conversion of an existing antigen-cognate Th17 population from a tolerizing-state to a cytotoxicity-inducing state, as would be expected after inoculation with an antigen that is recognized and presented by endogenous APCs in vivo. The association with TGFβ1 suggests that during antigen processing and presentation in vivo, TGFβ1 affected the immune response by promoting Th2 subpopulations and perhaps immunosuppressive Tregs.

In DCV-treated patients DCs were antigen-loaded ex vivo in the absence of suppressive cytokines (such as TGFβ1). After subcutaneous injection, it appears that the large dose of activated DCs produced sufficient amounts of IL12 to trigger a Th1 response. The first response likely came from cross-presentation of antigens directly to cytotoxic T lymphocytes (CTL) and/or from conversion of inherently plastic antigen-cognate Th17 cells into pro-cytotoxic states. It appears this triggering signal was only provided by ex vivo loaded DC leading to increases in IL17, IFNγ, and TNFα and was not observed in TCV-treated patients.

After this first wave of Th17 cytotoxic conversion, a second wave of helper population likely developed based on the positive association between IL12p70 and IL4 in both TCV-treated (ANOVA, p = 0.001) and DCV-treated patients (ANOVA, p = 0.005). This is consistent with de-novo antigen induction of a Th2 response, but in DCV-treated patients this was accompanied by increases in IFNγ (p = 0.001) and TNFα (p < 0.0001), which were not seen following TCV. The association of cytotoxic cytokines in DCV-treated patients suggests new helper cells sustained a persistent Th1-response, while in TCV-treated patients only a Th2-response persisted. Furthermore, in the DCV group there was a new Th2 response evidenced by PCA and DA of the immunoglobulins that showed a correlative increase and a class switching of IgG1 and IgG3 as opposed to IgG2, IgG4, and IgM. This was only observed in the DCV group, suggesting that while both treatment arms respond with a Th2 mechanism, the ex vivo-generated DCs may present antigens at better signal to noise ratio and thereby enhance or induce de-novo immunoglobulin responses.

IL12 is a cytokine produced by APC- when they are activated by exposure to antigens [40, 44]. In terms of antigen processing, the differences between the treatment arms is the source of the APCs: endogenous in situ APC in the case of TCV, and ex vivo antigen loaded DC in the case of DCV. In DCV the antigens are processed by DCs derived ex vivo from peripheral blood monocyte [9]. DCs mature and migrate to lymph nodes after exposure to antigen, where they contact effector T-cells, B-cells and natural killer cells (NKs) [40, 68]. Efficient presentation of the antigen is regulated by the interaction of the MHCs with T-cell receptors (TCR), and by regulatory and costimulatory connectors as well as by cytokine and chemokine signals [69, 75]. In addition to T-cell interaction, the antigen processing and presentation is modified by the state of the APCs. First there is a difference in the maturation stage of the APCs. Studies have shown that DC maturation is accompanied by a marked reorganization of endocytic compartments [76–78], and a concomitant inhibition of antigen uptake [79–83]. Antigen uptake and processing is limited to immature DCs, which contribute to the functional distinction between immature and mature DCs [82]. Endogenous APCs represent a minor population of cells at the site of TCV injections. In contrast the ex vivo loaded DCV injection contains a massive dose of 1 to 30 million immature DCs that originated from peripheral monocytes, that have not been exposed to additional maturation factors such as lipopolysaccharides. The phenotype and functionality of the ex vivo derived DCs is expected to be substantially different from the in situ APCs. As previously described, the immature DCs generated in vitro are more efficient for cross-presentation initially [83]. In addition to the massive number of simultaneously activated APCs (DCs) in DCV, the maturation differences could explain the initial Th1 cytotoxic response, cytotoxic conversion of Th17, followed by the Th2 response which could be mediated by endogenous APCs responding to apoptotic antigen-loaded DC, as opposed to the predominate Th2 response associated with the smaller number of more mature and less numerous APCs induced by TCV.

DCs are at the nexus of innate and adaptive immunity and have evolved to orchestrate a multi-pronged immune response [84]. Regardless of antigen source, DC are able to present antigen by both MHC I and MHC II pathways to induce both Th1 and Th2 immune responses [83–89], both of which are necessary for an optimal immune response to tumor antigen [90]. DC also can induce Th17 responses [91]. The role of Th17 cells in cancer is of increasing interest, especially because Th17 cells exhibit a plasticity that can result in their differentiation into Treg or Th17/Th1 cells [37, 92, 93]. The latter increasingly lose the ability to secrete IL17, but are able to secrete larger quantities of TNF, IL2, GM-CSF, and IFNγ than classical Th1 cells. It appears that TGFβ drives Th17 cells to become Tregs, and the absence of TGFβ and the presence of cytokines such as IL12 and IL23 is required for conversion to the Th17/Th1 phenotype. Although Th17 cells and Th17/Th1 cells are not believed to be cytotoxic, this Th1 helper phenotype is associated with increased cytotoxic T lymphocytes (CTL) in the tumor microenvironment. The ability of antigen-loaded DC to induce differentiation of Th17 cells into Th1 helper cells associated with anti-tumor effects was demonstrated in animal models [94]. Our analysis suggests that the patient-specific DCVs induced similar immune responses.

### Innate pathways

Pattern-recognition receptors (PRR) are proteins expressed by immune cells that recognize pathogen-associated molecular patterns as danger signals [95, 96]. IL6, IL8, and TNFα are produced in response to PRR that trigger a response from NK cells that is associated with production of IL15, IL18, and IFNγ [14–17]. Cytokines that regulate innate immunity are produced primarily by mononuclear phagocytes such as macrophages and DCs, although they can also be produced by T-lymphocytes, natural killer (NK) cells, endothelial cells, and mucosal epithelial cells. PRRs In injured tissue the innate immune system down-regulates effector mechanisms and restores homoeostasis via cytokines such as IL10 and TGFβ1 that are released by macrophages, preferentially the M2 subset, which can induce Tregs, inhibit pro-inflammatory cytokine production, and induce tissue healing by regulating extracellular matrix protein deposition and angiogenesis.

After treatment In the TCV arm, none of the changes in innate cytokines (pro-inflammatory or regulatory) correlated with overall survival or any of the survival subgroups. Th1/Th17 activity may be balancing a regulatory response through TGFβ1 and IL10. In the TCV arm, the lack of significant cytotoxic activity may tip the balance in favor of a regulatory mechanism. Linear regression of post-treatment changes in the NK-secreted cytokine IL15 revealed correlations with IFNγ (p < 0.001), IL2 (p < 0.001), IL5 (p < 0.001), IL6 (p < 0.001), IL7 (p < 0.001), IL8 (p < 0.001), IL10 (p < 0.001), IL13 (p < 0.001), IL17 (p < 0.001), IL21 (p = 0.009), IL23 (p = 0.002), IL12p70 (p < 0.001), TGFβ1 (p < 0.001), consistent with activation of the innate system.

Analysis of IL15 changes in the DCV arm showed a similar activation of the innate system including correlations with IFNγ (p = 0.002), IL2 (p = 0.005), IL5 (p = 0.001), IL6 (p < 0.001), IL7 (p < 0.001), IL8 (p < 0.001), IL10 (p = 0.01), IL13 (p < 0.022), IL17 (p < 0.001), IL12p70 (p < 0.002), TGFβ1 (p < 0.001), and TNFα (p = 0.016). The only difference between TCV and DCV arms is the correlation between changes in levels of IL15 and TNFα. In the DCV arm TNFα (p = 0.014), TGFβ1 (p = 0.027), and IL10 (p = 0.025) correlated with survival in patients who survived less than 60 months. Changes in TNFα, which is thought to be secreted by activated macrophages and T cells, correlated with increased IL12 in DCV-treated patients (Fig. 6c) but did not correlate with IL12 changes in TCV-treated patients (Fig. 6d). This also supports the hypothesis that conversion of the local inflammatory process from regulatory to cytotoxic occurred only in DCV-treated patients in a process that included Th17 and the innate immune system.

## Discussion

This study provides additional insight into differences in immune responses elicited by DCV and TCV. Both vaccines presented autologous tumor antigens but were associated with different immune responses and different survival benefit. While the results in the study are purely correlative, they are suggestive of underlying immunologic mechanisms of action. The major finding of this analysis is that DCV was associated with a multipronged immune response that included innate, Th2, and Th1/Th17 responses while the TCV immune responses were limited to innate and Th2. Direct correlation between Th1/Th17 changes and survival was also demonstrated. The results provide additional evidence that for therapeutic cancer vaccines it may be advantageous to present antigens by DC that were loaded with antigen ex vivo.

The analysis presented herein is complex and involved correlations of multiple variables derived from relatively small sample sizes. In addition to the analysis described in the manuscript, IBM SPSS (build 1.0.0.1298) was used to create a standard model using Automatic Linear Modeling function, targeting survival as an independent variable. A forward stepwise model was selected and AICC criteria, or F statistics (include effects with p < 0.05, remove effects at p > 0.1) was used for entry/removal of variables. Although the results were significant for many predictors, we felt that the reliability of the test might be criticized because of (small sample and large variability. Therefore, we performed a variable reduction with PCA and DA (variables normality verified) based on Bayesian estimation. Later, the synthetical variables obtained by PCA reduction of various groups of variables was examined in a regression model, but that approach did not provide meaningful results. The PCA graphics of the main components were presented herein without a statistical conclusion because representation of the differences between groups was provided with more confidence in the DA results. Nonetheless, these observations are hypothesis generating, or suggestive, clearly more cases would be needed for a robust statistical analysis.

With regard to immunoglobulins, there was evidence of a Th2 response that included increases in IgM in both treatment arms, especially in the DCV arm. The Th2 response was presumably mediated by endogenous, in situ DCs in the TCV-arm, while the ex vivo antigen-loaded DCV likely caused an early cytokine-mediated or cross-presentation response, followed by a new presentation of antigens through a typical helper pathway. In the TCV arm the changes are presumably in response to the additional antigenic stimulation provided by the irradiated tumors cells while in the DCV arm we believe that the initial cross presentation by DCV induced a new Th2 response as evidenced by immunoglobulin class switching. An association with a Th1 T-cell response is evidenced by the increase in IFNγ and TNFα with IL-12 only in the DCV arm, but this was not evident in the TCV arm.

Just prior to each subcutaneous injection, GM-CSF was admixed with tumor cells for TCV and dendritic cells for DCV for its adjuvant effects [97, 98], and specific effects on dendritic cells [99]. GM-CSF was given in the same dose and schedule in both arms; therefore, in the absence of a GM-CSF alone control arm, we cannot identify the specific effects that GM-CSF induced in each arm. GM-CSF was not one of the cytokines measured in the analysis; so, we have not data re its association with other cytokines in the principal component analyses. We know that levels of granulocyte colony stimulating factor (G-CSF) and macrophage colony stimulating factor (M-CSF) did not change after three injections of either TCV or DCV. Thymus and activation regulated chemokine CCL17 (TARC), which is induced by GM-CSF [100], was elevated significantly and similarly in both arms, and therefore excluded from the analysis.

The clinical trial from which this data was derived was the first randomized study testing therapeutic cancer vaccines in which there was a difference in survival in the treatment arms and for which associated proteomic data has been analyzed extensively for changes in, and correlations, with circulating markers [9]. Trying to decipher immune responses and their relation to clinical outcome in vaccine clinical trials is challenging. In one study in which colorectal cancer patients were treated with autologous dendritic cells loaded with allogeneic tumor cell lysate, plasma and serum samples were collected prior to vaccination and continuously during treatment [101]. Patients classified as having stable disease had increasing levels of IL2, IL5, TNFα, IFNγ, and GM-CSF while increases in carcinoembryonic antigen (CEA) and (TIMP-1) levels were associated with progressive disease. No correlative changes were noted for IL1b, IL4, IL6, IL8, IL10, IL12, macrophage inflammatory protein 1beta (MIP-1β), Interferon-inducible protein 10 (IP-10), or Eotaxin. That study was limited by the lack of a control arm and the limited number of cytokines examined. In another study, immune monitoring was conducted in association with an 815-patient six-arm trial that randomized patients with surgically resected stage 3 and 4 melanoma to peptide vaccines or placebo with GM-CSF or placebo in patients of appropriate HLA-type, and GM-CSF or placebo in patients who were HLA-A2 negative [102, 103]. One challenge for correlative analyses was that none of the treatment variables impacted overall survival compared to placebo [36]. The focus of the immune analysis was primarily on changes in immune cell phenotypes and their recognition of injected antigens rather than on changes in cytokines. There were no vaccine-specific correlations identified, and the cellular and humoral responses did not correlate with survival in the manner predicted [103].

The major strengths of our study include: (1) the use of data and samples from a randomized clinical trial that tested ATA presentation by two different cell sources (dendritic cells and cancer cells), (2) the availability of paired blood samples obtained at baseline and after 3 weekly injections, (3) the treatments tested were associated with different survivals, enabling a direct correlation between treatment, immune response, and survival, (4) the large number of immune markers tested, (5) the long-term follow-up for correlations with survival, and (6) the power of the statistical tools used to group positively and negatively correlated variables. The limitations of the study include: (1) the relatively small sample size, (2) paired samples were not available for 7.0% of the patients, (3) samples were not available for testing at earlier and later time points other than week-0 and week-4.

## Conclusions

**Fig. 7:**
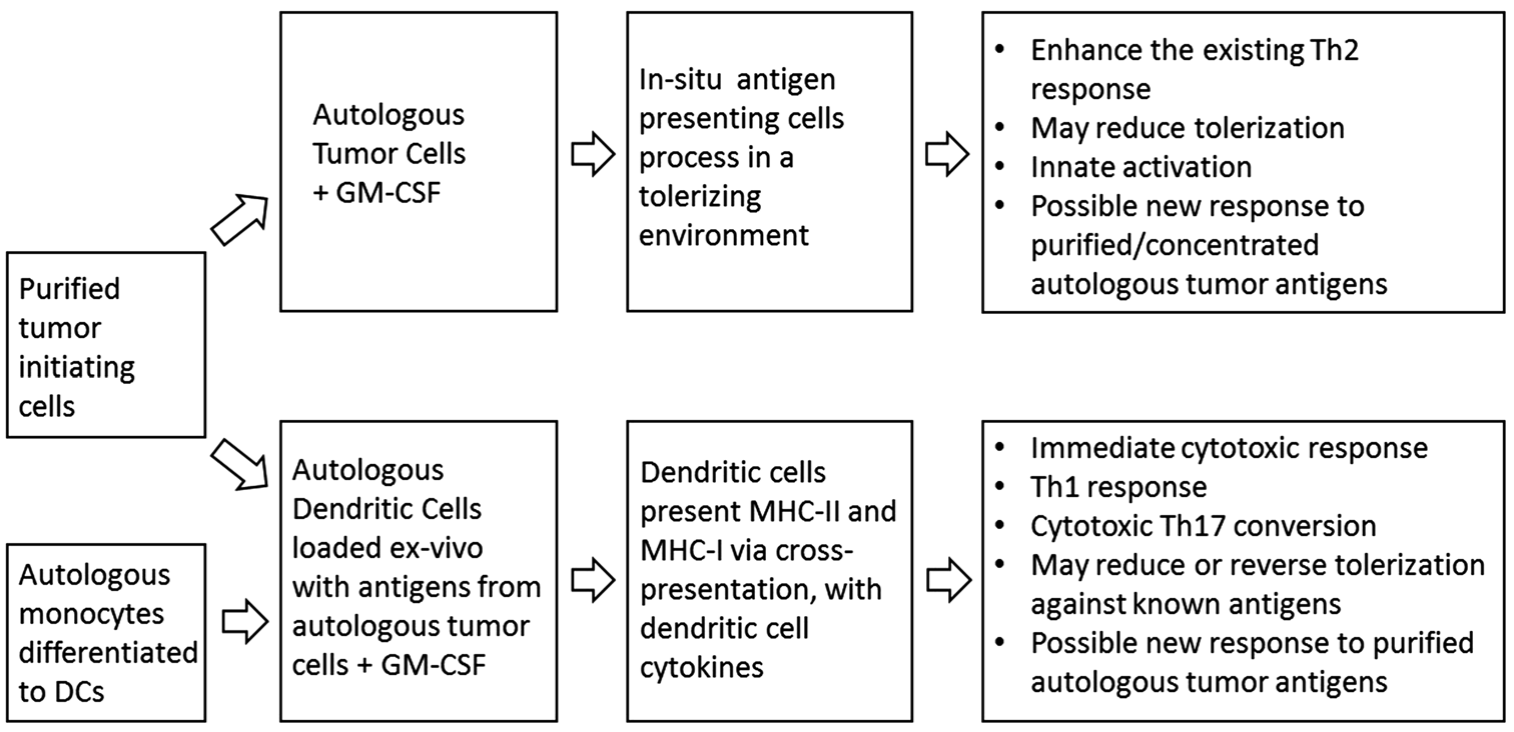
Infographic summary of findings

DCV induced a more effective immune response than that induced by TCV, and these immune responses were associated with improved survival. DCV was associated with innate, Th1/Th17, and Th2 responses while TCV was only associated with innate and Th2 responses. Figure 7 is an infographic summary of these changes.

## Supplementary information

**Additional file 1.** All patients: variance of main components for all patients at baseline.

**Additional file 2.** TCV-treated patients: variance of main components.

**Additional file 3.** TCV-treated patients: KMO and Bartlett’s Test.

**Additional file 4.** TCV-treated patients: Variance. Initial Eigenvalues are rotated with Varimax and Kaiser Normalization method.

**Additional file 5.** Variance explained.

**Additional file 6.** KMO and Bartlett’s Test.

**Additional file 7.** Variance. Initial Eigenvalues are rotated with Varimax with Kaiser Normalization method.

**Additional file 8.** Wilks’ Lambda test.

**Additional file 9.** First canonical discriminant function explained 90.2% of variance.

**Additional file 10.** Tests of equality of group means (univariate ANOVA).

**Additional file 11.** Wilks’ Lambda is significant for the first function.

**Additional file 12.** First 2 canonical discriminant functions explain 100% of variance.

**Additional file 13.** First 2 canonical discriminant functions used in the analysis explain 100% of variance.

**Additional file 14.** Wilks’ Lambda test of functions.

**Additional file 15.** Cox regression using IgM baseline values.

## Data Availability

The clinical and proteomic datasets analyzed during the current study are available from the corresponding author on reasonable request.

## Abbreviations

APC: Antigen presenting cell
ATA: Autologous tumor antigens
B2M: Beta2microglobulin
BTS: Bartlett’s Test of Sphericity
CEA: Carcinoembryonic antigen
CTL: Cytotoxic T lymphocytes
DC: Dendritic cell
DCV: Dendritic cell vaccine
DA: Discriminant analysis
FAS: FS-7-associated surface antigen (CD95)
GM-CSF: Granulocyte-macrophage colony stimulating factor
IFNγ: Interferon gamma
Ig: Immunoglobulin
IL: Interleukin
IP-10: Interferon-inducible protein 10 (CXCL10)
KMO: Kaiser–Meyer–Olkin Measure of Sampling Adequacy
MHC: Major histocompatibility complex
MIP-1 β: macrophage inflammatory protein 1beta (CCL4)
NK: Natural killer
PRR: Pattern-recognition receptors
PC: Principal component
PCA: Principal component analysis
PD-1: Programmed death protein-1
sPD-1: Soluble programmed death protein-1
TIMP-1: Tissue metalloproteinase inhibitor 1
TNFα: Tumor necrosis factor alpha
TGFβ: Transforming growth factor beta
Th: T lymphocyte helper
Treg: Regulatory T cell
TCV: Tumor cell vaccine
