# Supplementary material for: Cytokine network analysis of immune responses before and after autologous dendritic cell and tumor cell vaccine immunotherapies in a randomized trial
Source: J Transl Med. 2020 Apr 21;18:176. doi: 10.1186/s12967-020-02328-6 (PMC7171762; doi:10.1186/s12967-020-02328-6)
Supplement: Supplementary file 1 — Additional file 1. All patients: variance of main components for all patients at baseline. [file 12967_2020_2328_MOESM1_ESM.docx]

Additional files

Additional file 1. All patients: variance of main components for all patients at baseline.

| Component | Initial Eigenvalues >1.0 | | |
| --- | --- | --- | --- |
|  | Total | % of Variance | Cumulative % |
| 1 | 18.031 | 64.395 | 64.395 |
| 2 | 2.106 | 7.523 | 71.918 |
| 3 | 1.622 | 5.793 | 77.711 |
| 4 | 1.244 | 4.444 | 82.155 |
| 5 | 1.086 | 3.879 | 86.034 |
