# Supplementary material for: Cytokine network analysis of immune responses before and after autologous dendritic cell and tumor cell vaccine immunotherapies in a randomized trial
Source: J Transl Med. 2020 Apr 21;18:176. doi: 10.1186/s12967-020-02328-6 (PMC7171762; doi:10.1186/s12967-020-02328-6)
Supplement: Supplementary file 2 — Additional file 2. TCV-treated patients: variance of main components. [file 12967_2020_2328_MOESM2_ESM.docx]

Additional file 2. TCV-treated patients: variance of main components

| Component | Initial Eigenvalues | | |
| --- | --- | --- | --- |
|  | Total | % of Variance | Cumulative % |
| 1 | 12.828 | 41.382 | 41.382 |
| 2 | 3.842 | 12.392 | 53.775 |
| 3 | 3.394 | 10.948 | 64.723 |
| 4 | 2.050 | 6.613 | 71.336 |
| 5 | 1.512 | 4.877 | 76.214 |
| 6 | 1.416 | 4.568 | 80.782 |
| 7 | 1.281 | 4.133 | 84.915 |
