# Supplementary material for: Cytokine network analysis of immune responses before and after autologous dendritic cell and tumor cell vaccine immunotherapies in a randomized trial
Source: J Transl Med. 2020 Apr 21;18:176. doi: 10.1186/s12967-020-02328-6 (PMC7171762; doi:10.1186/s12967-020-02328-6)
Supplement: Supplementary file 5 — Additional file 5. Variance explained. [file 12967_2020_2328_MOESM5_ESM.docx]

Additional file 5. Variance explained

| Component | Initial Eigenvalues | | |
| --- | --- | --- | --- |
|  | Total | % of Variance | Cumulative % |
| 1 | 12.129 | 39.126 | 39.126 |
| 2 | 3.907 | 12.604 | 51.729 |
| 3 | 3.250 | 10.484 | 62.213 |
| 4 | 2.231 | 7.197 | 69.410 |
| 5 | 1.692 | 5.459 | 74.869 |
| 6 | 1.583 | 5.105 | 79.975 |
| 7 | 1.491 | 4.809 | 84.784 |
| 8 | 1.169 | 3.771 | 88.555 |
