# Supplementary material for: Cytokine network analysis of immune responses before and after autologous dendritic cell and tumor cell vaccine immunotherapies in a randomized trial
Source: J Transl Med. 2020 Apr 21;18:176. doi: 10.1186/s12967-020-02328-6 (PMC7171762; doi:10.1186/s12967-020-02328-6)
Supplement: Supplementary file 6 — Additional file 6. KMO and Bartlett’s Test. [file 12967_2020_2328_MOESM6_ESM.docx]

Additional file 6. KMO and Bartlett's Test.

| Kaiser-Meyer-Olkin Measure of Sampling Adequacy. | | .718 |
| --- | --- | --- |
| Bartlett's Test of Sphericity | Approx. Chi-Square | 277.385 |
|  | Df | 91 |
|  | Sig. | .000 |
