# Supplementary material for: Cytokine network analysis of immune responses before and after autologous dendritic cell and tumor cell vaccine immunotherapies in a randomized trial
Source: J Transl Med. 2020 Apr 21;18:176. doi: 10.1186/s12967-020-02328-6 (PMC7171762; doi:10.1186/s12967-020-02328-6)
Supplement: Supplementary file 7 — Additional file 7. Variance. Initial Eigenvalues are rotated with Varimax with Kaiser Normalization method. [file 12967_2020_2328_MOESM7_ESM.docx]

Additional file 7. Variance. Initial Eigenvalues are rotated with Varimax with Kaiser Normalization method.

| Component | Rotation Sums of Squared Loadings | | |
| --- | --- | --- | --- |
|  | Total | % of Variance | Cumulative % |
| 1 | 6.859 | 48.994 | 48.994 |
| 2 | 4.666 | 33.327 | 82.322 |
