# Supplementary material for: Cytokine network analysis of immune responses before and after autologous dendritic cell and tumor cell vaccine immunotherapies in a randomized trial
Source: J Transl Med. 2020 Apr 21;18:176. doi: 10.1186/s12967-020-02328-6 (PMC7171762; doi:10.1186/s12967-020-02328-6)
Supplement: Supplementary file 8 — Additional file 8. Wilks’ Lambda test. [file 12967_2020_2328_MOESM8_ESM.docx]

Additional file 8. Wilks' Lambda test

| Test of Function(s) | Wilks' Lambda | Chi-square | Df | Sig. |
| --- | --- | --- | --- | --- |
| 1 through 2 | .003 | 59.398 | 38 | .015 |
| 2 | .145 | 19.312 | 18 | .373 |
