# Supplementary material for: Cytokine network analysis of immune responses before and after autologous dendritic cell and tumor cell vaccine immunotherapies in a randomized trial
Source: J Transl Med. 2020 Apr 21;18:176. doi: 10.1186/s12967-020-02328-6 (PMC7171762; doi:10.1186/s12967-020-02328-6)
Supplement: Supplementary file 9 — Additional file 9. First canonical discriminant function explained 90.2% of variance. [file 12967_2020_2328_MOESM9_ESM.docx]

Additional file 9. First canonical discriminant function explained 90.2% of variance

| Function | Eigenvalue | % of Variance | Cumulative % | Canonical Correlation |
| --- | --- | --- | --- | --- |
| 1 | 54.074 | 90.2 | 90.2 | .991 |
| 2 | 5.897 | 9.8 | 100.0 | .925 |
