# Supplementary material for: Cytokine network analysis of immune responses before and after autologous dendritic cell and tumor cell vaccine immunotherapies in a randomized trial
Source: J Transl Med. 2020 Apr 21;18:176. doi: 10.1186/s12967-020-02328-6 (PMC7171762; doi:10.1186/s12967-020-02328-6)
Supplement: Supplementary file 10 — Additional file 10. Tests of equality of group means (univariate ANOVA). [file 12967_2020_2328_MOESM10_ESM.docx]

Additional file 10. Tests of equality of group means (univariate ANOVA)

|  | Wilks' Lambda | F | Sig. |
| --- | --- | --- | --- |
| Change_B2M | .996 | .039 | .962 |
| Change_Fas | .915 | .881 | .431 |
| Change_FasL | .939 | .618 | .549 |
| Change_IFNγ | .938 | .626 | .545 |
| Change_IgG1 | .996 | .035 | .966 |
| Change_IgG2 | .951 | .492 | .619 |
| Change_IgG3 | .840 | 1.811 | .191 |
| Change_IgG4 | .950 | .502 | .613 |
| Change_IgM | .941 | .596 | .561 |
| Change_IL2 | .839 | 1.825 | .188 |
| Change_IL3 | .920 | .829 | .452 |
| Change_IL4 | .922 | .800 | .464 |
| Change_IL5 | .933 | .679 | .519 |
| Change_IL6 | .952 | .483 | .624 |
| Change_IL7 | .945 | .549 | .586 |
| Change_IL8 | .892 | 1.152 | .337 |
| Change_IL10 | .944 | .568 | .576 |
| Change_IL13 | .873 | 1.381 | .275 |
| Change_IL15 | .883 | 1.265 | .305 |
| Change_IL17 | .936 | .644 | .536 |
| Change_IL18 | .936 | .647 | .535 |
| Change_IL21 | .977 | .228 | .798 |
| Change_IL22 | .992 | .075 | .928 |
| Change_IL23 | .952 | .482 | .625 |
| Change_IL27 | .910 | .941 | .408 |
| Change_IL12p40 | .884 | 1.246 | .310 |
| Change_IL12p70 | .908 | .958 | .402 |
| Change_PD1 | .844 | 1.752 | .200 |
| Change_TGFβ1 | .846 | 1.729 | .204 |
| Change_TNFα | .796 | 2.431 | .115 |
