# Supplementary material for: Cytokine network analysis of immune responses before and after autologous dendritic cell and tumor cell vaccine immunotherapies in a randomized trial
Source: J Transl Med. 2020 Apr 21;18:176. doi: 10.1186/s12967-020-02328-6 (PMC7171762; doi:10.1186/s12967-020-02328-6)
Supplement: Supplementary file 11 — Additional file 11. Wilks’ Lambda is significant for the first function. [file 12967_2020_2328_MOESM11_ESM.docx]

Additional file 11. Wilks' Lambda is significant for the first function

| Test of Function(s) | Wilks' Lambda | Chi-square | Df | Sig. |
| --- | --- | --- | --- | --- |
| 1 through 2 | .002 | 45.496 | 28 | .020 |
| 2 | .161 | 13.685 | 13 | .396 |
