# Supplementary material for: Cytokine network analysis of immune responses before and after autologous dendritic cell and tumor cell vaccine immunotherapies in a randomized trial
Source: J Transl Med. 2020 Apr 21;18:176. doi: 10.1186/s12967-020-02328-6 (PMC7171762; doi:10.1186/s12967-020-02328-6)
Supplement: Supplementary file 12 — Additional file 12. First 2 canonical discriminant functions explain 100% of variance. [file 12967_2020_2328_MOESM12_ESM.docx]

Additional file 12. First 2 canonical discriminant functions explain 100% of variance

| Function | Eigenvalue | % of Variance | Cumulative % | Canonical Correlation |
| --- | --- | --- | --- | --- |
| 1 | 68.503 | 92.9 | 92.9 | .993 |
| 2 | 5.201 | 7.1 | 100.0 | .916 |
