# Supplementary material for: Cytokine network analysis of immune responses before and after autologous dendritic cell and tumor cell vaccine immunotherapies in a randomized trial
Source: J Transl Med. 2020 Apr 21;18:176. doi: 10.1186/s12967-020-02328-6 (PMC7171762; doi:10.1186/s12967-020-02328-6)
Supplement: Supplementary file 13 — Additional file 13. First 2 canonical discriminant functions used in the analysis explain 100% of variance. [file 12967_2020_2328_MOESM13_ESM.docx]

Additional file 13. First 2 canonical discriminant functions used in the analysis explain 100% of variance.

| Function | Eigenvalue | % of Variance | Cumulative % | Canonical Correlation |
| --- | --- | --- | --- | --- |
| 1 | 1.268 | 73.1 | 73.1 | .748 |
| 2 | .467 | 26.9 | 100.0 | .564 |
