# Supplementary material for: Cytokine network analysis of immune responses before and after autologous dendritic cell and tumor cell vaccine immunotherapies in a randomized trial
Source: J Transl Med. 2020 Apr 21;18:176. doi: 10.1186/s12967-020-02328-6 (PMC7171762; doi:10.1186/s12967-020-02328-6)
Supplement: Supplementary file 14 — Additional file 14. Wilks’ Lambda test of functions. [file 12967_2020_2328_MOESM14_ESM.docx]

Additional file 14. Wilks' Lambda Test of Functions

| Test of Function(s) | Wilks' Lambda | Chi-square | df | Sig. |
| --- | --- | --- | --- | --- |
| 1 through 2 | .301 | 16.229 | 4 | .003 |
| 2 | .681 | 5.177 | 1 | .023 |
