# Supplementary material for: Cytokine network analysis of immune responses before and after autologous dendritic cell and tumor cell vaccine immunotherapies in a randomized trial
Source: J Transl Med. 2020 Apr 21;18:176. doi: 10.1186/s12967-020-02328-6 (PMC7171762; doi:10.1186/s12967-020-02328-6)
Supplement: Supplementary file 15 — Additional file 15. Cox regression using IgM baseline values. [file 12967_2020_2328_MOESM15_ESM.docx]

Additional file 15. Cox regression using IgM baseline values

|  | B | SE | Wald | df | Sig. | Exp(B) |
| --- | --- | --- | --- | --- | --- | --- |
| IgM in TCV | -.849 | .345 | 6.042 | 1 | 0.014 | .428 |
| IgM in DCV | -.254 | .480 | .281 | 1 | 0.596 | .776 |
